# The Role of Myocutaneous Flaps in the Treatment of Patients with Multiple Decubitus Ulcers

**DOI:** 10.25122/jml-2019-0104

**Published:** 2019

**Authors:** Catalin Gheorghe Bejinariu, Silviu Adrian Marinescu

**Affiliations:** Department of Plastic and Reconstructive Surgery, “Bagdasar-Arseni” Emergency Clinical Hospital, Bucharest, Romania

**Keywords:** decubitus ulcer, myocutaneous flap, tensor fascia lata muscle flap, sacral reconstruction, trochanteric reconstruction

## Abstract

The current research aims to present the therapeutic approach in the case of a paraplegic patient hospitalized in the Plastic Surgery Department at the “Bagdasar-Arseni” Emergency Clinical Hospital for the treatment of decubitus ulcers located at the level of the sacral, left trochanteric and posterior thoracic regions. The particularity of the case is given by the complexity of the surgical interventions necessary for the reconstruction of the above-mentioned anatomical regions. In order to cover the sacral region, two gluteal myocutaneous flaps were used, followed by a tensor fascia lata flap for the trochanteric lesion. For the thoracic defect, the surgical team has chosen the technique of excision and direct suture. Following reconstructive surgery, the patient had a favorable local evolution, being included in an intensive medical recovery program within the same health unit.

## Introduction

The decubitus ulcers that appear in paraplegic patients represent a serious health problem, imposing the adoption of a set of measures that aim at one hand to prevent their deepening, and on the other hand, to find a surgical solution that will speed up the healing process. In this way, it is possible to facilitate the faster inclusion of the patient in the intensive recovery program. Delaying reconstructive surgery can have devastating effects on the recovery and socio-professional integration of these patients. Another aspect to consider is the risk of sepsis in relation to these lesions. In the case of patients with limited mobility, the existence of cutaneous lesions presents a high risk of developing infections, especially those with antibiotic-resistant bacteria. The plastic surgeon’s armamentarium comprises an extensive range of therapeutic solutions, including local and free transferred flaps, which may represent the optimal solution in these cases.

The current paper presents the case of a 44-year-old patient, the victim of a road accident that resulted in complex vertebro-medullary trauma (T8-T9 dislocation), thoracic and abdominal trauma, multiple rib fractures, hemothorax, and right lung atelectasis. As a result of the trauma, the patient remained paraplegic and was admitted to the plastic surgery department for the treatment of the grade IV decubitus ulcers located at the level of the thoracic, sacral, and left trochanteric regions. During multiple hospitalizations, the patient was infected with antibiotic-resistant strains of Proteus mirabilis and Morganella morganii.

To solve this case, a therapeutic protocol was developed that involved the specific antibiotic treatment according to the antibiogram results associated with rebalancing of the patient and performing a series of reconstructive procedures during one surgical intervention that included: the coverage of the soft-tissue defect in the sacral region with gluteal flaps, the excision and the direct closure of the posterior thoracic lesion. One month after the first intervention, the surgical team performed the reconstruction with a tensor fascia lata flap for the trochanteric region. The patient was monitored for 18 months after the reconstructions, and the follow-up consults were performed every three months during this interval.

**Figure 1:**
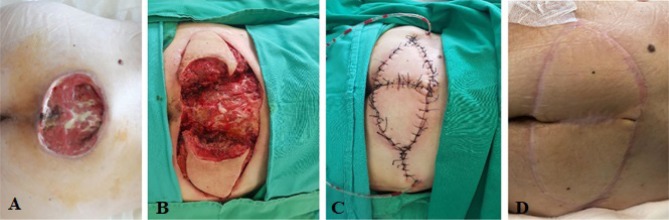
Reconstructive approach for the coverage of the sacral soft tissue defect. **A.** preoperative aspect; **B.** excision followed by flap dissection; **C.** reconstruction with gluteal myocutaneous flaps; **D.** postoperative aspect at 30 days

Following the reconstruction of the sacral region with gluteal myocutaneous flaps advanced to the median line, the local evolution was favorable, the aspiration drainage being removed five days after the surgery, and the suture threads being extracted 14 days after the intervention (Figure 1).

**Figure 2:**
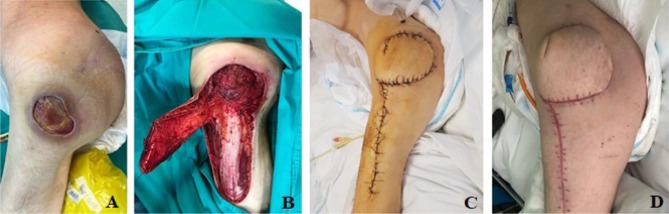
Reconstructive approach for the coverage of the trochanteric soft tissue defect. **A.** preoperative aspect; **B.** excision followed by flap dissection; **C.** reconstruction with a tensor fascia lata flap; **D.** postoperative aspect at 30 days

Regarding the coverage of the soft tissues defect located at the trochanteric region, the use of the tensor fascia lata flap proved to be a good solution, the local evolution being favorable, the drainage being removed five days after the surgery, followed by suture removal 14 days after the intervention (Figure 2).

**Figure 3:**
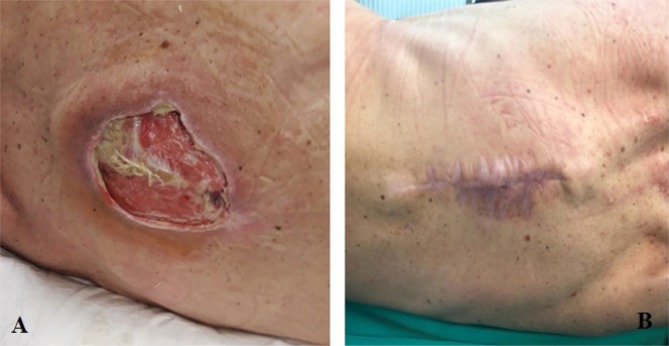
Reconstructive approach for the coverage of the thoracic pressure sore – excision and primary wound closure, followed by the coverage of the defect with a split-thickness skin graft. **A.** preoperative aspect; **B.** postoperative aspect at 30 days

In an initial stage, the thoracic pressure sore was excised, and the defect was covered by the advancement of the paramedian cutaneous flaps, but the increased tension on the primary suture line led to wound dehiscence. Subsequently, the delayed healing required the use of a small (1 cm^2^) free split-thickness skin graft harvested from the anterior aspect of the right thigh (Figure 3).

During the follow-up consults, the flaps were fully integrated, and no new pressure sores were detected.

## Discussion

The usage of myocutaneous flaps for the treatment of decubitus ulcers is a durable solution [1], the muscular component ensuring adequate coverage of the soft-tissue defect on the one hand, and on the other hand, the vascular inflow needed to accelerate the healing process and reduce the rate of relapse [2].

According to the specialized literature, the reconstruction with perforator flaps proves to be a durable solution for the coverage of the soft tissue defects after the excision of the pressure sores [3]; despite this fact, the myocutaneous flaps are considered a “workhorse” in the management of this type of lesions. The surgical protocol was chosen based on the patient’s pathological history, the increased number of lesions that required reconstruction, and the personal experience of the surgical team related to myocutaneous flap reconstructions.

The advancement of the gluteal myocutaneous flaps towards the median line in order to cover the remaining defect after the excision of the devitalized tissues begins with a triangular cutaneous incision, followed by dissection in anatomical planes and fascial sectioning to allow the advancement of the flap [4]. In order to achieve a sufficient mobilization of the flap, the lateral aspect of the triangle (corresponding to the apex of the triangle) is incised deep through the gluteal fascia and the surrounding tissue. Thereafter, the myocutaneous flaps are advanced medially to cover the soft-tissue defect and sutured into position in anatomical planes [5-8].

Concerning the tensor fascia flap, the main indication is the coverage of the soft-tissue defect located at the trochanteric level. After performing the excision of the devitalized tissues, it is essential to prepare the recipient area through a wide excision of all the pressure sore extensions and the granulation tissue. Respecting the preoperative drawing and having the tensor fascia lata as the primary reference, the flap is dissected and rotated in order to cover the affected region [9-12]. One detail is related to achieving the successful excision of the superficial skin layer in the pivot region in the axis of which the flap is rotated so as to limit its bulky (unpleasant) aspect.

Decubitus lesions located in the thoracic region require careful analysis of the therapeutic options [13-15]. The use of split-thickness skin grafts is rarely a solution in such cases due to the increased relapse rate. However, in this case, the defect was covered by advancing the cutaneous flaps, the skin graft being the solution for the dehiscence and the reduction of the healing time. The mirage of performing a complex reconstructive intervention can expose the patients to unjustified risks, given the relatively limited number of reconstructive options dedicated to this anatomical region [16-17]. The classical approach, consisting of the direct suture after advancing the local cutaneous tissue, proved to be the optimal solution for this patient [18]. However, the pressure sores located at this level require special attention in terms of appropriate therapeutic conduct.

## Conclusions

The gluteal myocutaneous flaps advanced to the median line represent a firm solution for covering the remaining defects after the excision of the large grade IV pressure sores, the significant muscle contribution helping to cover the defect and reducing the recurrence rate.

The reconstruction of the trochanteric region by using the tensor fascia flap is associated with a favorable local evolution in the conditions of an increased safety profile.

Excision and direct suture in the case of soft tissue defects located at the thoracic level may be the optimal solution in carefully selected cases.

In light of the presented results, in the case of patients with multiple pressure sores associated with infections with resistant bacteria, the specific antibiotic treatment followed by complex reconstructions with myocutaneous flaps may represent an efficient method of accelerating the recovery process.

## Conflict of Interest

The authors confirm that there are no conflicts of interest.
